# Dynamic Recyclable High-Performance Epoxy Resins via Triazolinedione–Indole Click Reaction and Cation–π Interaction Synergistic Crosslinking

**DOI:** 10.3390/polym16131900

**Published:** 2024-07-02

**Authors:** Ming He, Jing Li, Jiajing Xu, Lukun Wu, Ning Li, Shuai Zhang

**Affiliations:** Research Center of Laser Fusion, China Academy of Engineering Physics, Mianyang 621900, China; heming223591@163.com (M.H.); jingli0218@163.com (J.L.); jiajingxu@163.com (J.X.); lukingswu@foxmail.com (L.W.); lncaep@163.com (N.L.)

**Keywords:** click chemistry, cation–π, fluorescence response, recycling

## Abstract

Thermosetting plastics exhibit remarkable mechanical properties and high corrosion resistance, yet the permanent covalent crosslinked network renders these materials challenging for reshaping and recycling. In this study, a high-performance polymer film (EI_25_-TAD_5_-Mg) was synthesized by combining click chemistry and cation–π interactions. The internal network of the material was selectively constructed through flexible triazolinedione (TAD) and indole via a click reaction. Cation–π interactions were established between Mg^2+^ and electron-rich indole units, leading to network contraction and reinforcement. Dynamic non-covalent interactions improved the covalent crosslinked network, and the reversible dissociation of cation–π interactions during loading provided effective energy dissipation. Finally, the epoxy resin exhibited excellent mechanical properties (tensile strength of 91.2 MPa) and latent dynamic behavior. Additionally, the thermal reversibility of the C-N click reaction and dynamic cation–π interaction endowed the material with processability and recyclability. This strategy holds potential value in the field of modifying covalent thermosetting materials.

## 1. Introduction

Thermosetting materials have emerged as the preferred choice in the fields of electronics [1,2], coatings [3,4], sensors [5,6,7], adhesives [8,9,10], and aerospace [11], due to their outstanding mechanical properties, chemical resistance, and thermal stability. Nevertheless, the networks in traditional covalently crosslinked plastics are permanently fixed, exhibiting significantly limited ductility. Once these materials undergo a complete curing, reshaping and recycling become impractical. Epoxy materials, being the most widely utilized thermosetting resins, face similar challenges. Therefore, a designed epoxy material, with high performance, combining the advantages of thermosetting and thermoplastic plastics, is a subject that has ignited substantial interest among scientists.

Presently, the maturity of covalent dynamic chemistry has shattered the limitations on the inability to recycle and reprocess thermosetting materials, and these dynamically crosslinked structures are amicably referred to as Covalent Adaptive Networks (CANs) [12,13,14,15,16,17]. Dynamic covalent bonds can reversibly break and re-form under external stimuli such as temperature, light, pH, or chemical agents. This reversibility endows materials with self-healing capabilities, responsiveness, and processability while maintaining the stability and strength of the covalent bond structure. Building on this foundation, Luzuriaga et al. [18] assembled epoxy materials using disulfide bonds, resulting in materials exhibiting considerable mechanical performance while concurrently reducing the preparation cost of recyclable epoxy, showcasing significant potential in industrial applications. Zeng et al. [19] synthesized an epoxy material with a reversible boroxine bond network, demonstrating self-healing, welding, recyclability, and shape memory capabilities. Other epoxy CANs constructed using dynamic bonds, such as the Diels–Alder reaction [20,21], imine bonds [22,23,24], and ester exchange [25,26], have also exhibited commendable environmental friendliness.

When epoxy CANs are subjected to external loads, the preferential rupture of internal dynamic covalent bonds enhances toughness, although their strength generally remains. Researchers have attempted to introduce dynamic bonds to modify permanently crosslinked networks through both dynamic covalent bonds and non-covalent bonds [27,28,29,30]. The HGGelMA hydrogel developed by Wang et al. [31] comprised a weak non-covalent host–guest interaction integrated into a robust covalent network. The overall shape of the hydrogel is maintained by the covalent bonds and the non-covalent bonds, which can enhance its mechanical properties, facilitating rapid self-healing upon fracture. Zhao et al. [32] incorporated dynamic covalent boroxine bonds into a rigid epoxy material. The crosslinked network is jointly composed of a robust covalent structure and dynamic B-N bonds, resulting in a highly impressive mechanical performance. Further expanding on dynamic enhancements, Liu et al. [33] enhanced the mechanical properties of epoxidized natural rubber by grafting N-acetylglycine units, introducing hydrogen bonds that increase elasticity and toughness. This modification allows for reversible bond breaking under stress, which improves the material’s dynamic response to external loads as evidenced by the increased Young’s modulus, strength, and faster relaxation in highly grafted samples.

Triazolinedione–indole serves as an ideal combination of reversible and irreversible click reactions. The C-N bonds are sufficiently efficient, enabling the rapid completion of the reaction at room temperature without catalysts [34]. Du et al. [35] utilized a highly tough poly(methyl methacrylate) via a TAD–indole click reaction, which exhibited a force-induced reversibility at room temperature, as demonstrated by changes in the material fluorescence intensity. Hanay et al. [36] demonstrated the selective click chemistry of TAD–indole to prepare a hydrogel for drug delivery, showing significant potential applications in the medical field. Moreover, the cation–π interaction is a non-covalent dynamic strong interaction occurring between a cation and a conjugated π–electron cloud [37]. Zhou et al. [38] synthesized a recyclable epoxy resin with cation–π interactions, which has tension-triggered fluorescence changes and exhibited multiple memory effects. Chang et al. [39] described a supramolecular thermosetting plastic crosslinked by cation–π interactions, featuring the reversible installation and removal of cation–π bonds, as well as multiple stimuli-responsive properties.

**Figure 1:**
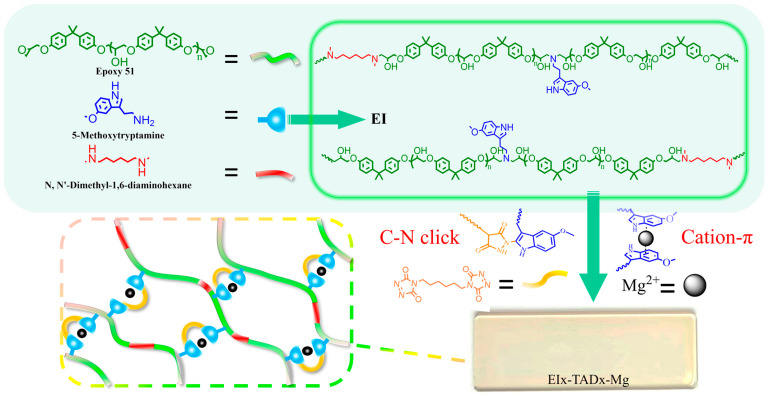
Synthesis of EI_25_-TAD*_x_*-Mg.

This study introduces a novel thermosetting epoxy material that combines a C-N click reaction with cation–π synergistic crosslinking, as illustrated in Figure 1. The internal C-N click within the polymer preserves the material’s rigid overall framework, ensuring structural integrity. Meanwhile, the weak reversible cation–π interactions constrict the covalent network, thereby not only enhancing the mechanical properties but also facilitating a unique adaptive response to environmental stresses. When subjected to external loads, the reversible dissociation of cation–π interactions further facilitates the effective energy dissipation within the epoxy network, thereby improving the toughness of the material under dynamic conditions. Consequently, the epoxy resin exhibited a superior mechanical performance, alongside responses to external triggers such as UV and fluorescence. Furthermore, this synergistic crosslinking network endows the material with reprocessability and recyclability.

## 2. Materials and Methods

### 2.1. Materials

Epoxy E51, 5-methoxytryptamine (98%), *N*,*N*′-dimethyl-1,6-hexanediamine (99%), ethyl carbazate (99%), hexamethylene diisocyanate (99%), hydrochloric acid (37%), nitric acid (65–68%), tetrahydrofuran (99.9%), magnesium sulfate anhydrous (99.5%), ethanol (99.8%), dichloromethane (99%, DCM), magnesium chloride (99%), and *N*,*N*-dimethylformamide (99%, DMF) were obtained from Aladdin Chemistry Co., Ltd. (Shanghai, China). The synthesis of hexamethylene bis-triazolinedione (HMBT) was executed following established protocols from previous studies. Other organic reagents required no further purification. Distilled water was produced in-house for use in the laboratory.

### 2.2. Synthesis and Characterization of HMBT

#### 2.2.1. Synthesis of Hexamethylene Bis-Semicarbazides

**Figure 2:**
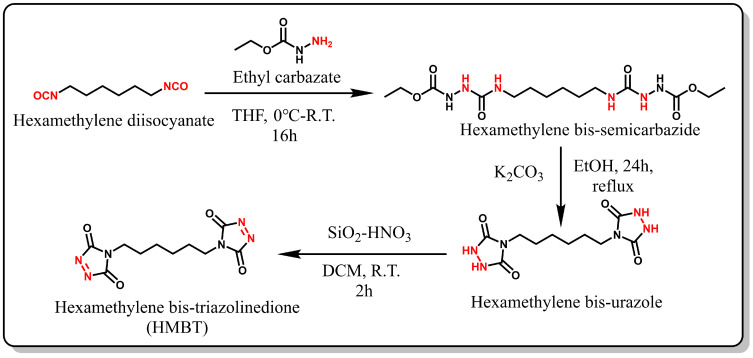
Synthesis of HMBT.

Under a nitrogen atmosphere, 10.04 g (96.4 mmol) of ethyl carbazate was dissolved in 60 mL of anhydrous tetrahydrofuran (THF) and transferred to a 250 mL round-bottom flask. Subsequently, an ice-cold solution of 8.11 g (48.2 mmol) of hexamethylene diisocyanate in 60 mL THF was added dropwise to the reaction mixture and stirred at 0 °C for 10 min. The resulting suspension was stirred for an additional 2 h, followed by filtration and multiple washings with THF. The semicarbazide was then vacuum-dried (yield: 97%). ^1^H NMR (400 MHz, DMSO-*d*_6_) data are as follows: δ (ppm) = 8.54 (d, *J* = 182.3 Hz, 1H), 7.56 (s, 1H), 6.30 (s, 1H), 4.01 (q, *J* = 7.1 Hz, 2H), 2.97 (dd, *J* = 12.9, 6.5 Hz, 2H), 1.34 (d, *J* = 6.0 Hz, 2H), 1.21 (s, 2H), 1.16 (t, *J* = 6.9 Hz, 3H) (see Figure 2).

#### 2.2.2. Synthesis of Hexamethylene Bis-Urazole

A suspension was prepared by dissolving 10 g (26.6 mmol) of amino urea in 300 mL of anhydrous ethanol under continuous stirring. Next, 12.4 g (90 mmol) of anhydrous potassium carbonate was added, and the mixture was heated to reflux and stirred continuously for 24 h. Subsequently, the solution was filtered to remove any residues, and the solid crude product was dissolved in ice-cold distilled water. Concentrated hydrochloric acid was then added dropwise at 0 °C to adjust the solution’s pH to 1. The resulting precipitate was filtered and washed with excess ice-cold distilled water. Finally, the white solid was dried under vacuum (yield: 30%). ^1^H NMR (400 MHz, DMSO-*d*_6_) data are as follows: δ (ppm) = 10.05 (s, 1H), 3.30 (d, *J* = 7.1 Hz, 1H), 1.55-1.41 (m, 1H), 1.22 (s, 1H).

#### 2.2.3. Synthesis of Hexamethylene Bis-Triazolinedione (HMBT)

Silica nitric acid was prepared according to a previously published method [40]. In a 40 mL glass vial, 3.2 g SiO_2_ was combined with 5 mL of concentrated nitric acid, and the mixture was stirred with a glass rod for 15 min. The resulting silica nitric (SiO_2_-HNO_3_) was washed with DCM and then vacuum-filtered. A suspension was prepared by adding 1.2 g SiO_2_-HNO_3_ and 0.5 g hexamethylenediamine to DCM, and the mixture was stirred for 2 h at room temperature, yielding a bright pink solution. The solution was then filtered, and anhydrous MgSO_4_ was added to the filtrate and mixed for at least 10 min. Then, we filtered the crude product, and MgSO_4_ was washed with DCM for three times. The solution was evaporated by using a rotary evaporator at 25 °C to prevent product degradation. Finally, the product was stored under nitrogen at −18 °C (yield: 50%). ^1^H NMR (400 MHz, DMSO-*d*_6_) data are as follows: δ (ppm) =3.46 (t, *J* = 7.1 Hz, 1H), 1.55 (s, 1H), 1.29 (s, 1H).

### 2.3. The Preparation of Epoxy Polymer EI_x_-TAD

**Figure 3:**
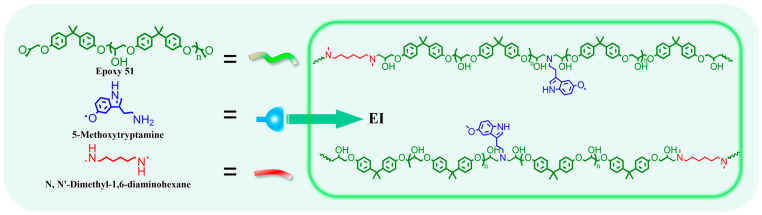
Synthesis of EI.

Epoxy E51(1 eq), 5-methoxytryptamine (0.25 eq), and *N*,*N*′-dimethyl-1,6-hexanediamine (0.75 eq) were dissolved in DMF and transferred to a three-neck flask. The reaction proceeded at 85 °C for 24 h (refer to Figure 3 and Table 1). The solution concentration was adjusted by DMF. And the polymer solution was dried at 80 °C for 12 h to remove the solvent; EI films are obtained. For EI*_x_*-TAD, as an example of EI_25_-TAD: An equal volume of DMF solution containing HMBT (0.125 eq) was mixed with the EI_25_ solution (1 eq). The mixture was rapidly stirred at low temperature to ensure thorough crosslinking of the prepolymer. The solution was rapidly cast onto slides and dried at 80 °C for 12 h to remove the solvent; homogeneous films of EI_25_-TAD are obtained. The film was subsequently immersed in water to facilitate detachment, followed by a water removal. Details of the experimental parameters are provided in Table S1. 

### 2.4. Preparation of Epoxy Polymer EI_25_-TAD_x_-Mg

The synthesis method for EI_25_-TAD is identical to that described in Section 2.3. After obtaining the EI_25_-TAD solution, MgCl_2_ was added and rapidly stirred at low temperature in 30 min to ensure complete dissolution of the solid, as detailed in Table 2. The polymer solution was promptly transferred to slides and dried at 80 °C for 12 h to remove the solvent; homogeneous films of EI_25_-TAD*_x_*-Mg are obtained. Subsequently, the film was immersed in water to facilitate detachment, followed by a water removal. 

### 2.5. Characterization

^1^H NMR were conducted on a superconducting nuclear magnetic resonance spectrometer, with chemical shifts calibrated using DMSO-*d*_6_. Infrared spectroscopy (FT-IR) was acquired in the form of KBr pellets using a Fourier-transform infrared spectrometer. Fluorescence spectrums (FL) were recorded using a fluorescence spectrophotometer with an excitation wavelength of 365 nm and slit widths of 5 nm (excitation)/5 nm (emission), covering an emission spectrum range from 400 to 700 nm. The characterization of the sample materials was conducted using a UV–Vis–NIR spectrophotometer, performing scans at a medium speed across the wavelength range of 200–800 nm. Thermal gravimetric (TG) analysis was performed using a thermal analyzer under a nitrogen atmosphere, with a heating speed of 10 °C/min within a temperature range of 20–800 °C. In a nitrogen atmosphere, differential scanning calorimetry (DSC) analysis was conducted using a differential scanning calorimeter at a scanning speed of 10 °C/min and a nitrogen flow rate of 200 mL/min, within a temperature range of 20–300 °C. Uniaxial tensile tests were performed using a universal testing machine at a rate of 2 mm/min. Each concentration sample film was tested three times to eliminate randomness. Energy-dispersive X-ray spectroscopy (EDS) of the samples were carried out using a field emission scanning electron microscope.

In situ relaxation-UV test: The film was fixed using a clamp and subjected to a load, with its UV absorption spectrum measured while in a relaxed state. Similarly, in the in-situ relaxation-FL test: By securing the sample film in a specialized fixture and applying a load, the fluorescence spectrum of the film in a relaxed state was measured.

## 3. Results and Discussion

### 3.1. Preparation and Characterizations of Materials

**Figure 4:**
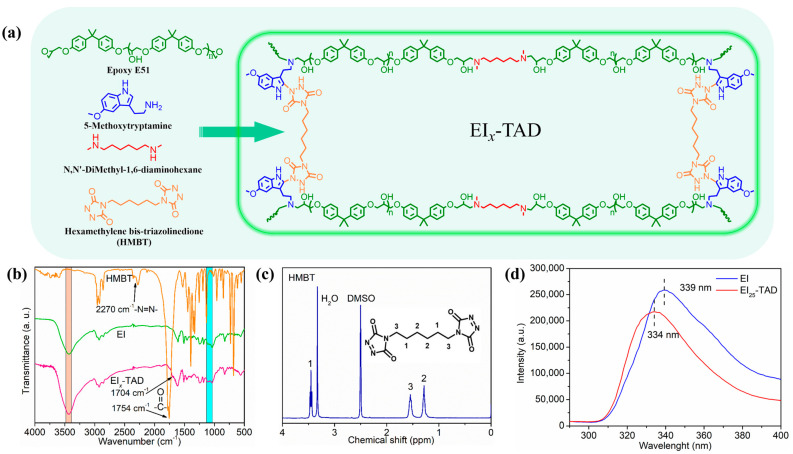
(**a**) Synthesis of EI*_x_*-TAD. (**b**) FT-IR spectrum of HMBT, EI, and EI*_x_*-TAD. (**c**) ^1^H NMR spectrum of HMBT. (**d**) Fluorescence spectrum of uncrosslinked and crosslinked films.

In this study, linear epoxy polymers functionalized with indole groups (EI) were synthesized through a simple one-pot method using epoxy E51, 5-methoxytryptamine, and *N*,*N*-dimethyl-1,6-hexanediamine, as illustrated in Figure 3 and Table S1. By varying the ratio of 5-methoxytryptamine to *N*,*N*-dimethyl-1,6-hexanediamine, different levels of indole side chains were incorporated. Subsequently, HMBT was added in proportion to the indole content, maintaining a molar ratio of HMBT to indole at 1:2, as detailed in Table 1 and Figure 4a. The characterization of HMBT was conducted using ^1^H NMR and FT-IR spectroscopy, as shown in Figure 4b,c. The FT-IR spectrum exhibited significant peaks at 2270 cm^−1^ and 1754 cm^−1^ corresponding to the -N=N- group and the carbonyl group (C=O), respectively [35]. 

The formation of the epoxy resin (EI*_x_*) was confirmed by an FT-IR analysis of the E51-type epoxy monomer and epoxy film (Figure 4b and Figure S1). The peaks in the 3400–3500 cm^−1^ range are due to the stretching vibrations of the hydroxyl and amine groups. The absence of characteristic peaks for epoxy groups in the 1080–1160 cm^−1^ range indicates that the epoxy group’s have fully reacted.

Following the introduction of HMBT into the EIx polymer, a click-crosslinked epoxy film (EI*_x_*-TAD) was obtained. The FT-IR spectrum of EI*_x_*-TAD is shown in Figure 4b. Compared to the EI polymer, it exhibits sharper and larger peaks within the 3400–3500 cm^−1^ range. The alteration is due to an increased content of -NH groups in the EI*_x_*-TAD film by the click chemistry reaction. A new peak observed at 1704 cm^−1^ is characteristic of the carbonyl group in ketones, formed by the stretching vibration of the carbon–oxygen double bond. Similar to EIx, characteristic peaks for epoxy groups were not observed in the infrared spectrum of EIx-TAD. Additionally, peaks indicative of -N=N- bonds were not detected. These results further confirm the completion of the click reaction and the effective preparation of EI*_x_*-TAD.

**Figure 5:**
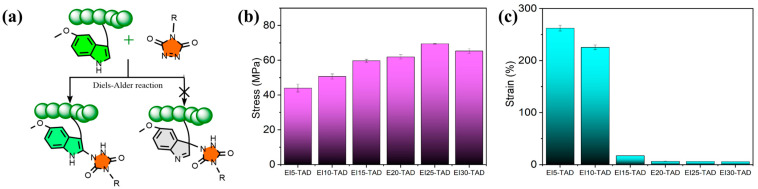
(**a**) Schematic diagram of chemical click site. (**b**) Tensile strength and (**c**) toughness curves of EI*_x_*-TAD.

Upon comparison of the fluorescence spectrum, it was observed that the fluorescence curve of the EI_25_-TAD experiences a blue shift, accompanied by a slight decrease in fluorescence intensity. This phenomenon is ascribed to the transfer of electron clouds during the click reaction (Figure 4d) [35,41]. The fluorescence of the crosslinked epoxy resin remains pronounced, indicating that the click reaction did not compromise the aromaticity of indole. The click site is identified as the 2-position of indole, preserving the aromatic structure while simultaneously establishing a more robust C-N crosslinking network (Figure 5a). The tensile strength and fracture toughness of the epoxy resin varies with changes in the concentration of TAD–indole, as shown in Figure 5b,c. The highest intensity of the sample is observed at 25%, with a fracture strength of 69.5 ± 0.3 MPa and a fracture strain of 6.1 ± 0.1%.

**Figure 6:**
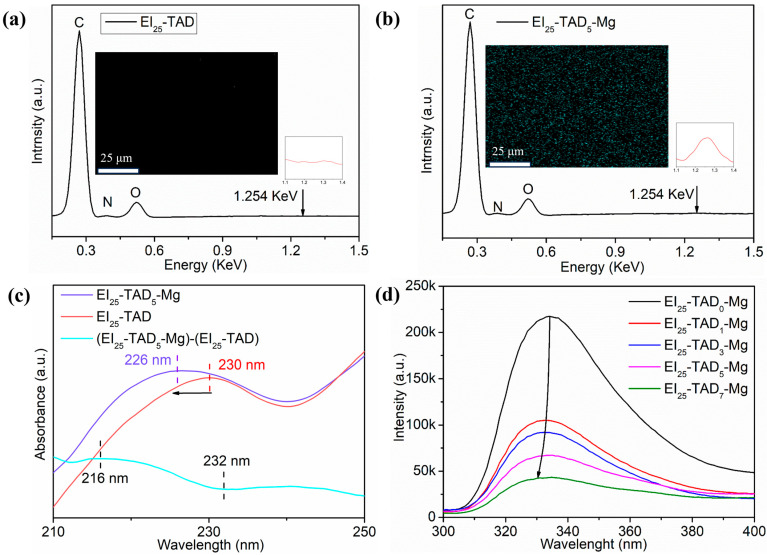
(**a**) EDS and mapping of EI_25_-TAD. (**b**) EDS and mapping of EI_25_-TAD_5_-Mg. (**c**) UV–Vis spectrum of EI_25_-TAD, and EI_25_-TAD_5_-Mg, and the difference spectrum between EI_25_-TAD_5_-Mg and EI_25_-TAD. (**d**) Fluorescence spectrum of EI_25_-TAD*_x_*-Mg.

The optimal TAD–indole concentration (25%) was utilized as the template for introducing cations. By incorporating magnesium chloride, Mg^2+^ were introduced into the polymer material. Cation–π interactions between metal ions and indole groups effectively condense the material’s network structure. Confirmation of the film preparation was accomplished through FT-IR, as shown in Figure S2. A notable contrast was observed between the homogeneous dispersion of Mg^2+^ within the EI_25_-TAD_5_-Mg and the lack of Mg^2+^ in EI_25_-TAD, as evident from the results of energy-dispersive X-ray spectroscopy in Figure 6a,b.

Subsequently, UV–visible spectrophotometry and fluorescence spectrophotometry were deployed to confirm the presence of cation–π interactions in EI_25_-TAD_5_-Mg [42]. Figure 6c illustrates the ultraviolet absorption spectrum of EI_25_-TAD and EI_25_-TAD_5_-Mg, along with the difference spectrum (EI_25_-TAD_5_-Mg)-(EI_25_-TAD). The formation of cation–π interactions led to a blue shift in the ultraviolet peak, moving from 230 nm to 226 nm, as evidenced by the negative intensity changes between 216 nm and 232 nm in the spectrum [43]. Furthermore, the cation–π interactions between Mg^2+^ and indole in EI_25_-TAD_5_-Mg prompted an electron cloud transfer towards Mg^2+^, resulting in a reduction in fluorescence intensity and a blue shift in the fluorescence curve, as observed in the fluorescence spectrum (Figure 6d) [38]. 

### 3.2. Mechanical Properties

**Figure 7:**
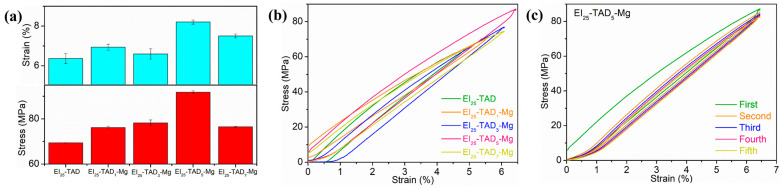
(**a**) The tensile properties of EI_25_-TAD*_x_*-Mg. (**b**) The cyclic strain–stress curves of EI_25_-TAD*_x_*-Mg. (**c**) The cyclic stress–strain curves of EI_25_-TAD_5_-Mg with five cycles.

The impact of the dynamic non-covalent bond content on the mechanical properties of EI_25_-TAD is depicted in Figure 7a. The results show that the inclusion of cation–π interactions enhances the material’s flexibility and strength. With the establishment of cation–π interactions, the epoxy films demonstrated remarkable improvements in mechanical properties. Notably, when 5% of the indole–TAD crosslinking sites in the material’s internal network form cation–π interactions, the sample’s mechanical performance reaches its peak. Specifically, the tensile strength of the material increased from 69.5 ± 0.3 MPa to 91.8 ± 0.6 MPa, and the fracture strain improved from 6.1 ± 0.1% to 8.2 ± 0.1%. To study the energy absorption efficiency of EI_25_-TAD*_x_*-Mg, cyclic stress–strain tests were conducted on films with different amount of Mg^2+^. As shown in Figure 7b, the tests stretched to 80% of the intrinsic fracture strain at a speed of 1 mm/min. The results showed that all films’ cyclic stress–strain curves exhibited hysteresis, indicating that the cation–π interactions within the films were continuously breaking and reforming, to dissipate the energy. In multiple cyclic tensile tests, the films containing Mg^2+^ retained their responsiveness to external mechanical stimuli, as shown in Figure 7c.

### 3.3. Scalable Network and Recycling

**Figure 8:**
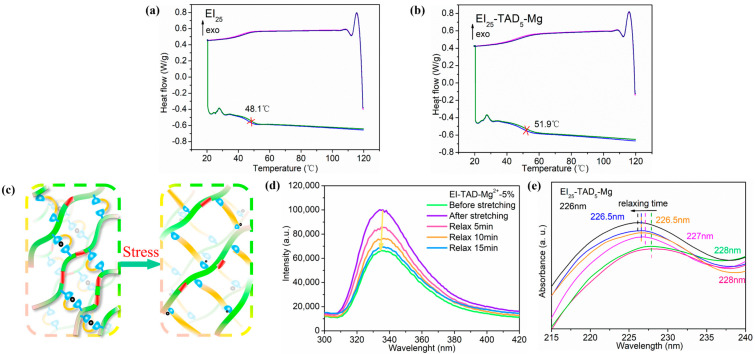
The second and third heating DSC curves for (**a**) EI_25_-TAD_5_-Mg and (**b**) EI_25_-TAD. (**c**) Schematic diagram of the film under external force. (**d**) In situ tensile-fluorescence mapping of EI_25_-TAD_5_-Mg. (**e**) In situ tensile-UV mapping of EI_25_-TAD_5_-Mg.

After the C-N crosslinked network was contracted by cation–π interactions, the material was tested by DSC. Figure 8a,b, respectively, show the second and third heating DSC curves for EI_25_ and EI_25_-TAD_5_-Mg. Compared to the uncrosslinked EI_25_ film, the introduction of a synergistic crosslinking network increased the glass transition temperature (Tg). The Tg of the film increased from 48.1 °C to 51.9 °C. This enhancement is primarily attributed to the TAD–indole crosslinking points, which restrict the mobility of the polymer chains. Additionally, cation–π interactions further tighten the network structure, ultimately elevating the material’s Tg [44].

As shown in Figure 8c, the EI_25_-TAD_5_-Mg film is subjected to external force stimulation, resulting in the disruption of the cation–π interactions’ network structure to break. Point-to-plane interactions are disrupted to dissipate energy, thereby inducing the migration of Mg^2+^ under continuous force. The Mg^2+^ engaged in new “point-to-plane” cation–π interactions when met with another click crosslinking point. To substantiate the film’s dynamic process, in situ tensile-fluorescence and in situ tensile-UV experiments were conducted. Under the influence of external forces, the disturbance of cation–π structures resulted in an increase in the film’s fluorescence intensity, as exemplified in Figure 8d, concomitant with a redshift in the fluorescence spectrum. As the film continues to relax, new cation–π interactions formed within the film, progressively reducing the fluorescence intensity, ultimately reinstating the film to its initial state. Similarly, under sustained force, the disruption of cation–π interactions led to a redshift, marked by the emergence of an ultraviolet absorption peak at 228 nm, as displayed in Figure 8e. As the duration of constant force increased, the ultraviolet absorption peak reverted to 226 nm. These results demonstrated that EI_25_-TAD_5_-Mg possesses a reversible motion behavior in response to external force stimuli in the fluorescence and UV spectrum. The reversible phenomenon of the material bestows it with significant research value in the field of sensors for signal transmission.

**Figure 9:**
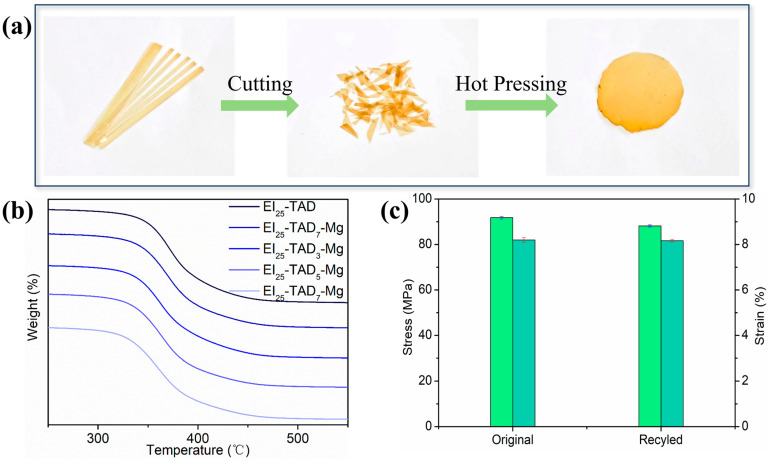
(**a**) Process of hot pressing. (**b**) TGA curves of EI_25_-TAD*_x_*-Mg. (**c**) Stress–strain curves of original and remodeling EI_25_-TAD_5_-Mg sample.

The reaction between TAD and indole exemplifies an ideal interplay between irreversible and reversible C-N reactions; the thermal reversibility of the C-N structure enables the recyclability of the material [34]. Additionally, cation–π interactions, a robust non-covalent bonding mechanism, ensure the material’s structural integrity while preserving its recyclability. The resulting network, effectively crosslinked through the synergistic interaction of click chemistry reactions and cation–π forces, demonstrates both the high mechanical properties and potential recyclability of the material. The dual crosslinking strategy effectively maintains the material’s thermal stability, as evidenced by the thermal decomposition temperature of the film. As recorded in Figure 9b, the thermal decomposition temperature of the material is 323.3 °C. To further investigate the recyclability of the EI25-TAD5-Mg film, the fragmented epoxy film was reprocessed at a temperature of 140 °C with a pressure of 30 MPa for a duration of 10 min (Figure 9a).

Subsequent tensile testing of the reprocessed films, as depicted in Figure 9c, showed that EI_25_-TAD_5_-Mg retained both its original mechanical properties and recovered its performance capabilities. The hot-pressed films exhibited a high tensile strength of 88.1 ± 0.5 MPa and a fracture strain of 8.1 ± 0.1%. Notably, the mechanical strength of the recycled films was restored to 96.6% of their original value, demonstrating the effectiveness of the synergistic crosslinking network in preserving material properties across multiple life cycles. Materials with an exceptional recovery performance hold tremendous potential in sustainable manufacturing practices.

## 4. Conclusions

This study proposed the synthesis of recyclable epoxy films by integrating a covalent thermally reversible click reaction and non-covalent cation–π crosslinking. EI_25_-TAD_5_-Mg demonstrates an excellent mechanical performance, which shows a 31% increase in tensile strength (91.6 ± 0.6 MPa) and a 36% increase in fracture strain (8.2 ± 0.1%) compared with EI_25_-TAD. Crosslinked networks exhibit responsiveness to stress stimuli through variations in fluorescence and ultraviolet curves during stretching, thereby elucidating the positive influence of dynamic bonding on stress responses. Furthermore, the processability and recyclability of this material have been enhanced, with a significant recovery efficiency manifested by the mechanical strength of the regenerated films, which attain 96.6% (88.5 ± 0.4 MPa) of their original strength. This strategy holds profound implications for the development of innovative high-performance recyclable materials within the domain of sustainable development.

## Figures and Tables

**Table 1:** The detailed receipt of EI*_x_*-TAD.

Samples	E51	5-Methoxytryptamine	*N*,*N*′-Dimethyl-1,6-hexanediamine	HMBT
EI_5_-TAD	10 mmol	0.5 mmol	9.5 mmol	0.25 mmol
EI_10_-TAD	10 mmol	1.0 mmol	9.0 mmol	0.50 mmol
EI_15_-TAD	10 mmol	1.5 mmol	8.5 mmol	0.75 mmol
EI_20_-TAD	10 mmol	2.0 mmol	8.0 mmol	1.00 mmol
EI_25_-TAD	10 mmol	2.5 mmol	7.5 mmol	1.25 mmol
EI_30_-TAD	10 mmol	3.0 mmol	7.0 mmol	1.50 mmol

**Table 2:** The detailed receipt of EI_25_-TAD*_x_*-Mg.

Samples	EI25-TAD	MgCl_2_
EI25-TAD	1 eq	0.000 eq
EI25-TAD1-Mg	1 eq	0.005 eq
EI25-TAD3-Mg	1 eq	0.015 eq
EI25-TAD5-Mg	1 eq	0.025 eq
EI25-TAD7-Mg	1 eq	0.035 eq

## Data Availability

The data are contained within the article.

## Supplementary Materials

The following supporting information can be downloaded at: https://www.mdpi.com/article/10.3390/polym16131900/s1, Table S1: The detailed receipt of EI_x_.; Figure S1: FT-IR spectrum of E51; Figure S2. FT-IR spectra of EI_25_-TAD_5_-Mg; Figure S3: ^1^H NMR spectrum of Hexamethylene Bis-Semicarbazides; Figure S4: ^1^H NMR spectrum of Hexamethylene Bis-Urazole; Figure S5: ^1^H NMR spectrum of Hexamethylene Bis-Triazolinedione (TAD).
