# Supplementary material for: Dynamic Recyclable High-Performance Epoxy Resins via Triazolinedione–Indole Click Reaction and Cation–π Interaction Synergistic Crosslinking
Source: Polymers (Basel). 2024 Jul 2;16(13):1900. doi: 10.3390/polym16131900 (PMC11243886; doi:10.3390/polym16131900)

Supporting Information

Dynamic recyclable high-performance epoxy resins via triazolinedione-indole click reaction and cation-π interaction synergistic crosslinking

Ming He, Jing Li, Jiajing Xu, Lukun Wu, Ning Li and Shuai Zhang*

Research Center of Laser Fusion, China Academy of Engineering Physics, Mianyang 621900, People's Republic of China

Corresponding authors:

Shuai Zhang,

Research Center of Laser Fusion, China Academy of Engineering Physics, Mianyang 621900, People's Republic of China

Email: zhangshuai_scu@126.com (S. Zhang)

The Preparation of Epoxy Polymer EI and the Film

Epoxy E51, 5-methoxytryptamine, and N, N'-dimethyl-1,6-hexanediamine were dissolved in DMF and transferred to a three-neck flask. The reaction proceeded continuously at 85°C for 24 hours. The sample solution concentration was adjusted by adding a total of 30 mL of DMF. Subsequently, the resulting solution was cast onto a Slides and dried at 80°C in an oven overnight to completely remove the DMF. After natural cooling to room temperature, the glass slides along with the films were immersed in deionized water for 3 hours to facilitate film separation. Following this, the film was carefully peeled off and dried at 90°C in an oven to remove water.

Table S1. The detailed receipt of EI_x.

Samples	Epoxy E51	5-methoxytryptamine	N, N'-dimethyl-1,6-hexanediamine
EI_5	3.92 g	0.095 g	1.37 g
EI_{10}	3.92 g	0.190 g	1.30 g
EI_{15}	3.92 g	0.285 g	1.23 g
EI_{20}	3.92 g	0.380 g	1.16 g
EI_{25}	3.92 g	0.475 g	1.08 g
EI_{30}	3.92 g	0.570 g	1.01 g

Figure S1. FT-IR spectrum of E51

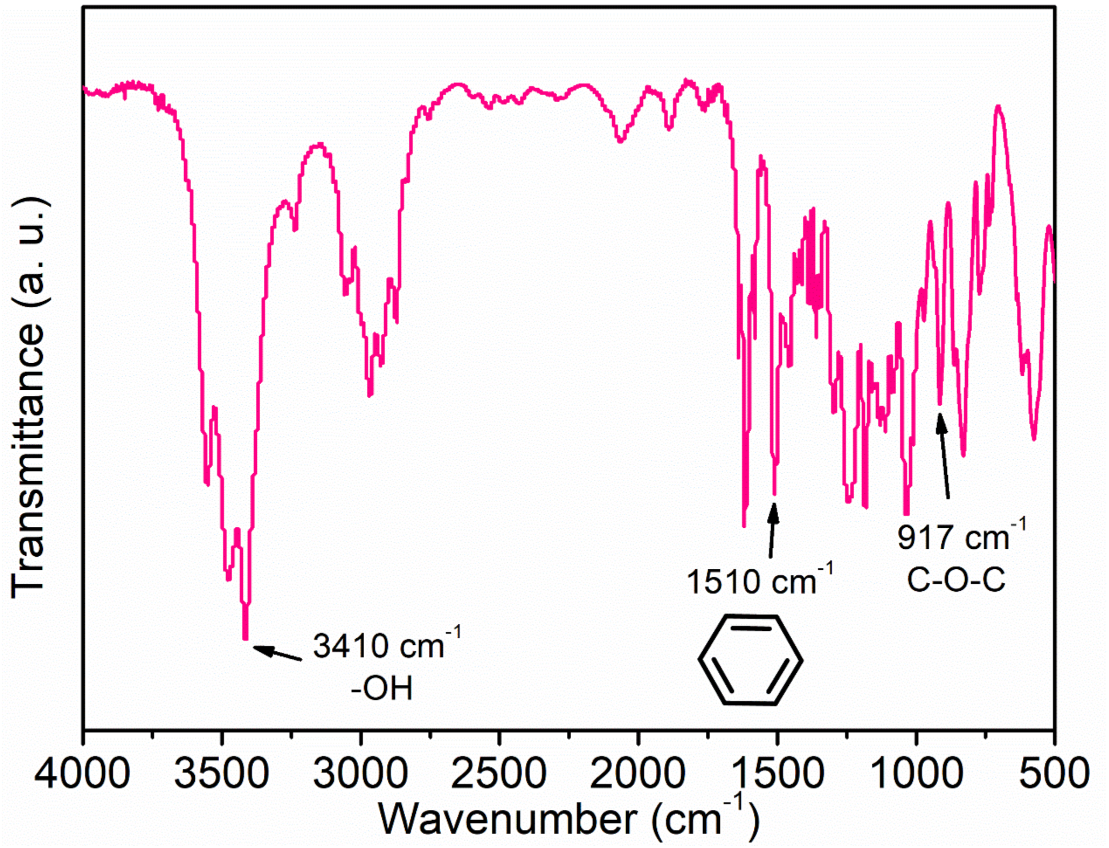

Figure S2. FT-IR spectra of EI_{25}-TAD_5-Mg.

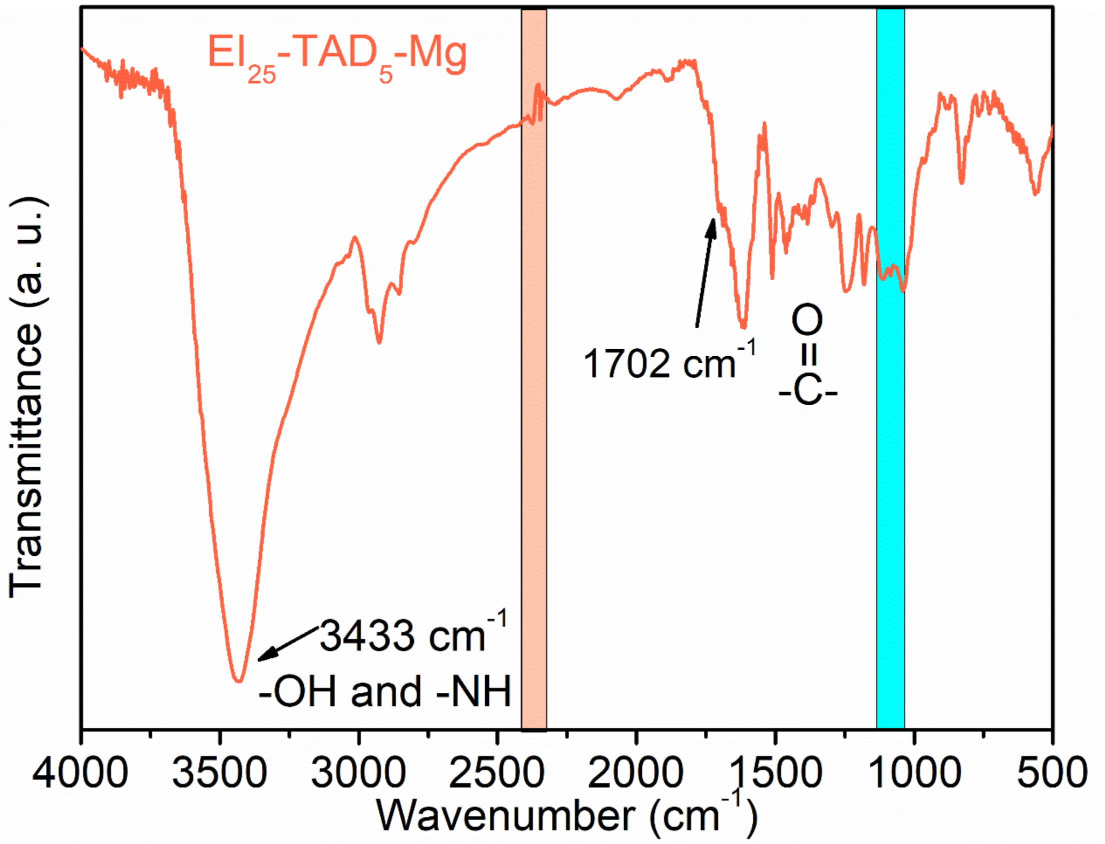

Figure S3. ^{1}H NMR spectrum of Hexamethylene Bis-Semicarbazides

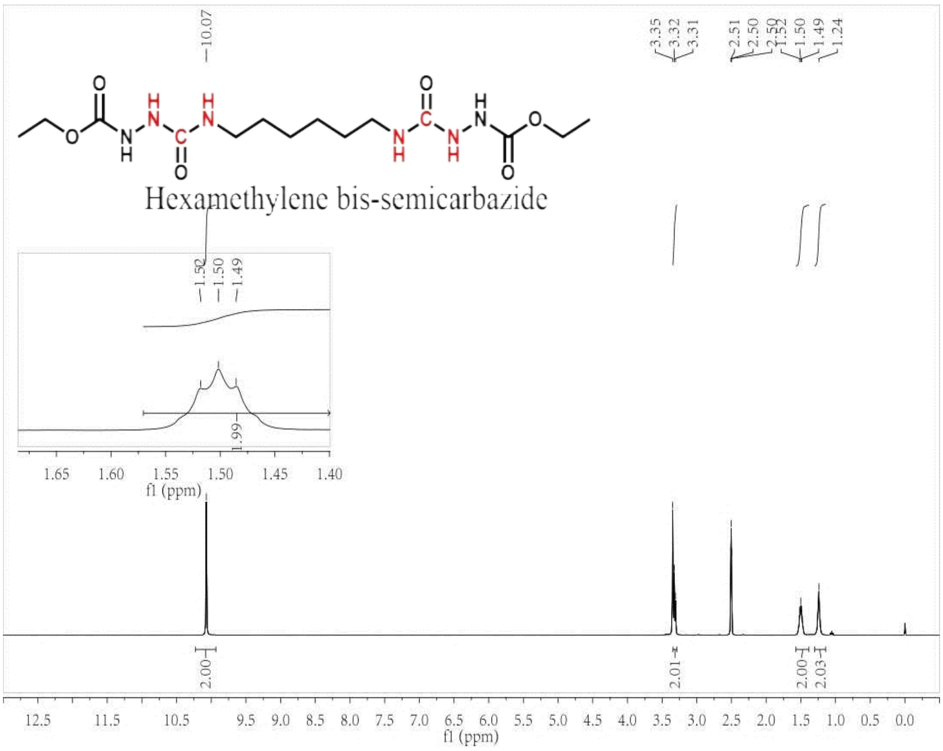

Figure S4. ^{1}H NMR spectrum of Hexamethylene Bis-Urazole

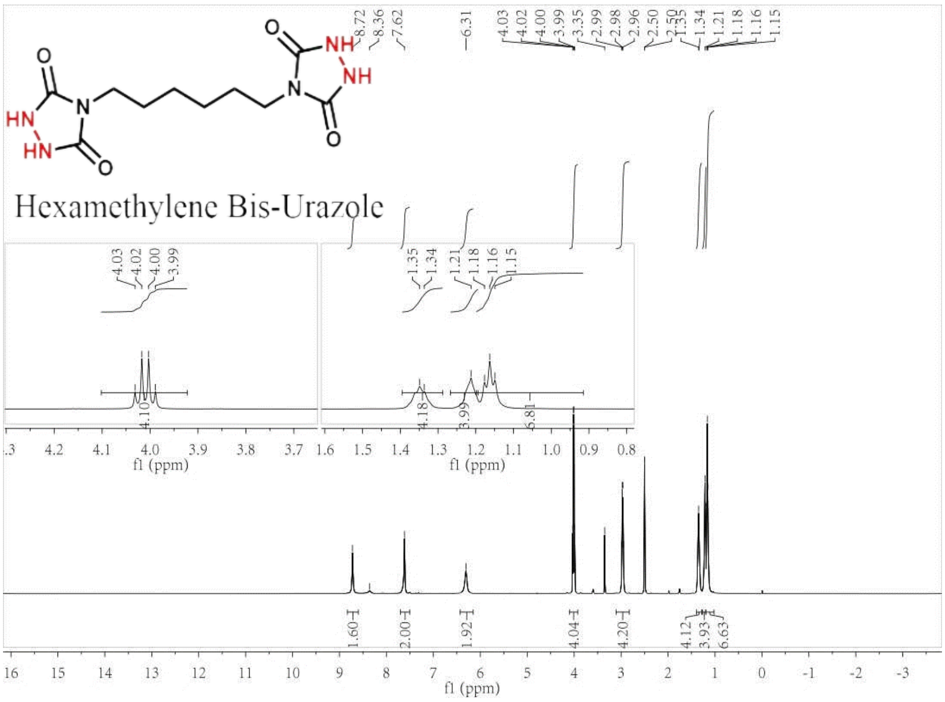

Figure S5. ^{1}H NMR spectrum of Hexamethylene Bis-Triazolinedione (TAD)

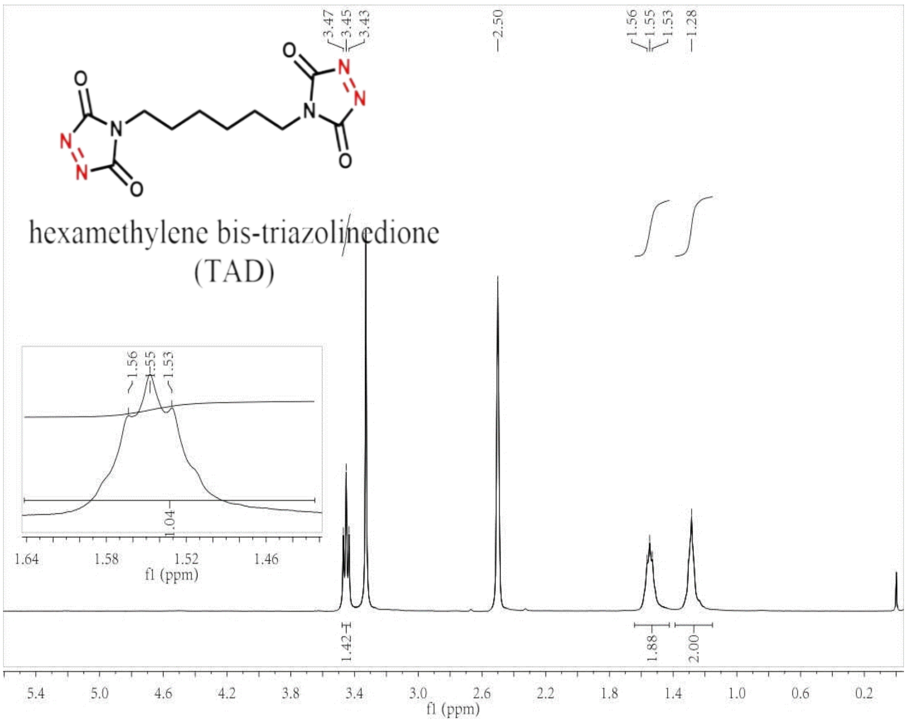

Supplement: Supplementary file 1 [file polymers-16-01900-s001.zip › polymers-2930960-supplementary.pdf]
